# Supplementary material for: Divergent Heat Stress Responses in Bactrocera tryoni and Ceratitis capitata
Source: Insects. 2024 Sep 30;15(10):759. doi: 10.3390/insects15100759 (PMC11508621; doi:10.3390/insects15100759)
Supplement: Supplementary file 1 [file insects-15-00759-s001.zip › insects-3139109-supplementary.pdf]

Figure S1 Average RIN number of each RNA library.

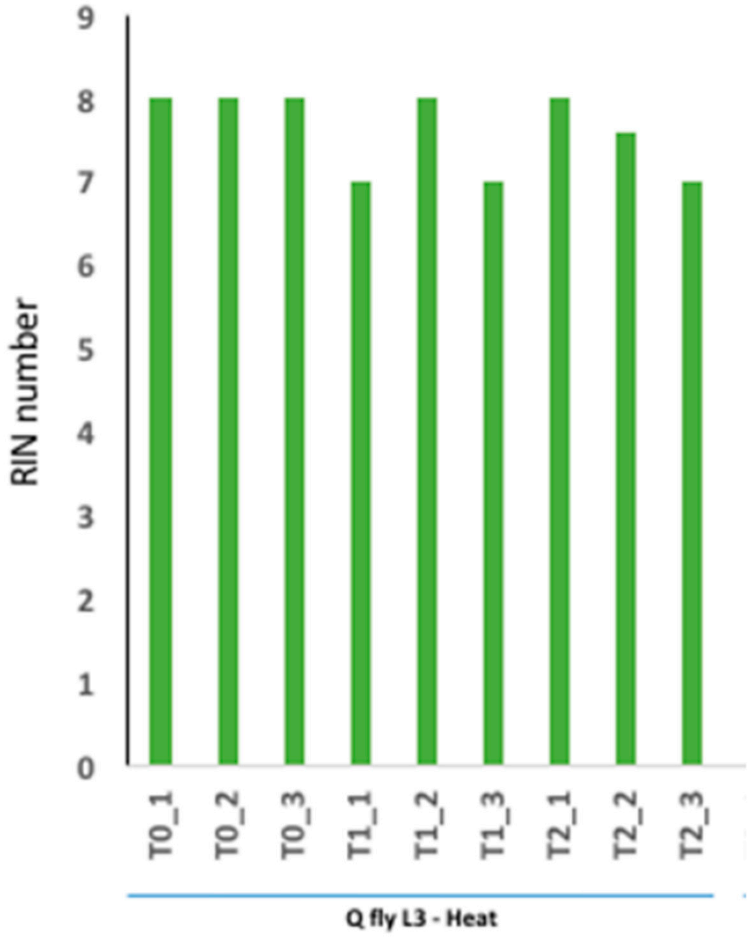

Table S1 Cq value for each library for barcoding.

**Library**	**Cq value**		
	R1	R2	R3
**Heat T0**	20	17	20
**Heat T1**	18	20	20
**Heat T2**	20	20	21
**Cold T0**	20	20	19
**Cold T1**	20	21	20
**Cold T2**	19	20	20

Table S2 Primers used for RNA sequencing.

Primer name	Primer sequence (5' – 3')
**First strand cDNA synthesis**	
**Bio_TS_RNA**	Biotin – CAG GAC GCT GTT CCG TTC AAT GGG
**RT_Hex**	CAG ACG TGT GCT CTT CCG ATC T NNN NNN
**Barcoding Optimisaton qPCR**	
**TS_qPCR**	CAG GAC CCT GTT CCG TTC AAT GGG
**RT_Hex qPCR**	CAG ACG TGT GCT CTT CCG ATC T
**Barcoding sequence**	
**i5_1**	AATGATACGGCGACCACCGAGATCTACAC [ATCCGGTA] ACACTTCGCTACAGGACGCTGTTCCGTTCAATGGG
**i5_2**	AATGATACGGCGACCACCGAGATCTACAC [TAGGCCAT] ACACTTCGCTACAGGACGCTGTTCCGTTCAATGGG
**i5_3**	AATGATACGGCGACCACCGAGATCTACAC [ACAACCTC] ACACTTCGCTACAGGACGCTGTTCCGTTCAATGGG
**i5_4**	AATGATACGGCGACCACCGAGATCTACAC [TGTTGGAG] ACACTTCGCTACAGGACGCTGTTCCGTTCAATGGG
**i5_5**	AATGATACGGCGACCACCGAGATCTACAC [TTCTCTCC] ACACTTCGCTACAGGACGCTGTTCCGTTCAATGGG
**i5_6**	AATGATACGGCGACCACCGAGATCTACAC [AAGAGAGG] ACACTTCGCTACAGGACGCTGTTCCGTTCAATGGG
**i5_7**	AATGATACGGCGACCACCGAGATCTACAC [GGTGCTAA] ACACTTCGCTACAGGACGCTGTTCCGTTCAATGGG
**i5_8**	AATGATACGGCGACCACCGAGATCTACAC [CCACGATT] ACACTTCGCTACAGGACGCTGTTCCGTTCAATGGG
**i7_A**	CAAGCAGAAGACGGCATACGAGAT [CGAATTGC] GTGACTGGAGTTCAGACGTGTGCTCTTCCGATCT
**i7_B**	CAAGCAGAAGACGGCATACGAGAT [GCTTAACG] GTGACTGGAGTTCAGACGTGTGCTCTTCCGATCT
**i7_C**	CAAGCAGAAGACGGCATACGAGAT [CACCAAGA] GTGACTGGAGTTCAGACGTGTGCTCTTCCGATCT
**i7_D**	CAAGCAGAAGACGGCATACGAGAT [GTGGTTCT] GTGACTGGAGTTCAGACGTGTGCTCTTCCGATCT
**i7_E**	CAAGCAGAAGACGGCATACGAGAT [GGAGAGAA] GTGACTGGAGTTCAGACGTGTGCTCTTCCGATCT
**i7_F**	CAAGCAGAAGACGGCATACGAGAT [CCTCTCTT] GTGACTGGAGTTCAGACGTGTGCTCTTCCGATCT
**i7_G**	CAAGCAGAAGACGGCATACGAGAT [TTGTAGCC] GTGACTGGAGTTCAGACGTGTGCTCTTCCGATCT
**i7_H**	CAAGCAGAAGACGGCATACGAGAT [AACATCGG] GTGACTGGAGTTCAGACGTGTGCTCTTCCGATCT
**Custom read 1**	ACACTTCGCTACAGGACGCTGTTCCGTTCAATGGG
**Custom index 2**	ACACTTCGCTACAGGACGCTGTTCCGTTCAATGGG

Table S3 Supplement table index read for RNA sequencing.

Barcode name	HiSeq index read	NextSeq index read
**i5_1**	ATCCGGTA	TACCGGAT
**i5_2**	TAGGCCAT	ATGGCCTA
**i5_3**	ACAACCTC	GAGGTTGT
**i5_4**	TGTTGGAG	CTCCAACA
**i5_5**	TTCTCTCC	GGAGAGAA
**i5_6**	AAGAGAGG	GGAGAGAA
**i5_7**	GGTGCTAA	TTAGCACC
**i5_8**	CCACGATT	AATCGTGG
**i7_A**	CGAATTGC	GCAATTCG
**i7_B**	GCTTAACG	CGTTAAGC
**i7_C**	CACCAAGA	TCTTGGTG
**i7_D**	GTGGTTCT	AGAACCAC
**i7_E**	GGAGAGAA	TTCTCTCC
**i7_F**	CCTCTCTT	AAGAGAGG
**i7_G**	TTGTAGCC	GGCTACAA
**i7_H**	AACATCGG	CCGATGTT

Table S4. Genes differentially expressed in Qfly L3 in response to heat treatment. T1 vs T0 are genes differentially expressed immediately after the heat treatment. T2 vs T0 are genes differentially expressed 2 hr after the heat treatment. Because *B. tryoni* genome is still not fully annotated, we used a close fly species, *B. dorsalis* to perform the query.

T1 vs T0	Gene Number	Gene Description	Fold Change
down	XM_019991234.1	high mobility group protein DSP1 (LOC105228822), transcript variant X3, mRNA	0.001
down	XM_018949369.1	mediator of RNA polymerase II transcription subunit 8 (LOC108978864), mRNA	0.001
down	XM_019991817.1	activating transcription factor 3 (LOC105230342), transcript variant X6, mRNA	0.001
down	XM_011208001.2	zinc finger protein 729-like (LOC105228255), transcript variant X2, mRNA	0.001
down	XM_019991770.1	unconventional myosin-XV (LOC105230352), transcript variant X3, mRNA	0.001
down	XM_018940259.1	uncharacterized LOC108973189 (LOC108973189), mRNA	0.002
down	XM_019989049.1	ras-specific guanine nucleotide-releasing factor RalGPS1 (LOC105222418), mRNA	0.008
down	XM_011205240.2	ATP synthase subunit d, mitochondrial (LOC105226383), transcript variant X3, mRNA	0.010
up	XM_014232231.1	cuticle protein 16.5, isoform B-like (LOC106615861), mRNA	8.712
up	XM_011200974.2	cytosolic 10-formyltetrahydrofolate dehydrogenase (LOC105223295), mRNA	8.984
up	XM_011214395.2	nucleolin 1 (LOC105232633), transcript variant X1, mRNA	9.653
up	XM_011214603.1	extensin (LOC105232778), mRNA	14.935

up	XM_019992338.1	ferritin subunit (LOC105231771), mRNA	48.641
up	XM_011199023.2	suppressor protein SRP40-like (LOC105221884), mRNA	210.905
up	XM_011212823.2	putative uncharacterized protein DDB_G0277255 (LOC105231502), transcript variant X5, mRNA	232.305
up	XM_011203253.2	ADP,ATP carrier protein (LOC105224974), transcript variant X2, mRNA	298.864
up	AJ535756.1	ovoA gene for zinc finger transcription factor	557.025
up	XM_019992608.1	glycogen-binding subunit 76A (LOC105232625), transcript variant X12, mRNA	599.474
up	XM_011205015.2	probable salivary secreted peptide (LOC105226196), mRNA	690.525
up	XM_018937116.1	G patch domain and ankyrin repeat-containing protein 1 homolog (LOC108971217), mRNA	712.325
up	XM_011204766.2	transmembrane protein 47 (LOC105226030), transcript variant X2, mRNA	778.859
up	XM_011206322.2	CUE domain-containing protein 1 (LOC105227115), transcript variant X2, mRNA	785.233
up	XM_011201216.2	serine-rich adhesin for platelets-like (LOC105223479), mRNA	807.350
up	XM_011199982.2	cyclin-dependent kinase inhibitor 1C (LOC105222592), mRNA	865.933
up	XM_011210902.2	zinc finger protein 236-like (LOC105230244), mRNA	901.126
up	XM_018946991.1	facilitated trehalose transporter Tret1-2 homolog (LOC108977365), mRNA	930.377
up	XM_019989335.1	protein new-glue 3-like (LOC109579384), mRNA	994.584

up	XR_852644.1	uncharacterized LOC105227775 (LOC105227775), ncRNA	1355.044
up	XM_011209471.2	NADH dehydrogenase [ubiquinone] 1 beta subcomplex subunit 10 (LOC105229289), mRNA	1802.797
up	XM_018940765.1	pollen-specific leucine-rich repeat extensin-like protein 1 (LOC108973475), mRNA	1942.340
up	XM_011200734.2	NEDD8 (LOC105223109), mRNA	1995.807
up	XM_014240037.1	ELAV-like protein 2 (LOC106621269), mRNA	2469.784
up	XM_011203012.2	epidermal growth factor receptor kinase substrate 8-like protein 2 (LOC105224802), transcript variant X1, mRNA	3554.091
**T2 vs T0**			
down	XM_014240037.1	ELAV-like protein 2 (LOC106621269), mRNA	0.0002
down	XM_019989335.1	protein new-glue 3-like (LOC109579384), mRNA	0.0004
down	XM_011203253.2	ADP, ATP carrier protein (LOC105224974), transcript variant X2, mRNA	0.0005
down	XM_011209471.2	NADH dehydrogenase [ubiquinone] 1 beta subcomplex subunit 10 (LOC105229289), mRNA	0.0007
down	XM_019992608.1	glycogen-binding subunit 76A (LOC105232625), transcript variant X12, mRNA	0.0007
down	XM_011200734.2	NEDD8 (LOC105223109), mRNA	0.0008
down	XM_011203012.2	epidermal growth factor receptor kinase substrate 8-like protein 2 (LOC105224802), transcript variant X1, mRNA	0.0011
down	XM_011210902.2	zinc finger protein 236-like (LOC105230244), mRNA	0.0014

down	XM_011204766.2	transmembrane protein 47 (LOC105226030), transcript variant X2, mRNA	0.0016
down	AJ535756.1	ovoA gene for zinc finger transcription factor	0.0016
down	XM_011205015.2	probable salivary secreted peptide (LOC105226196), mRNA	0.0019
down	XM_018940765.1	pollen-specific leucine-rich repeat extensin-like protein 1 (LOC108973475), mRNA	0.0020
down	XM_018946991.1	facilitated trehalose transporter Tret1-2 homolog (LOC108977365), mRNA	0.0021
down	XM_011201216.2	serine-rich adhesin for platelets-like (LOC105223479), mRNA	0.0021
down	XM_018937728.1	proline-rich extensin-like protein EPR1 (LOC108971567), mRNA	0.0060
down	XM_011212823.2	putative uncharacterized protein DDB_G0277255 (LOC105231502), transcript variant X5, mRNA	0.0092
down	XM_018935330.1	flocculation protein FLO11-like (LOC108970144), transcript variant X8, mRNA	0.0162
down	XM_011214603.1	extensin (LOC105232778), mRNA	0.0690
down	XM_014232231.1	cuticle protein 16.5, isoform B-like (LOC106615861), mRNA	0.1054
down	XM_011205478.2	alpha-tocopherol transfer protein-like (LOC105226558), mRNA	0.1902
up	XM_018943619.1	heat shock protein 68 (LOC108975260), mRNA	10.520
up	XM_018943606.1	heat shock protein 68-like (LOC108975252), mRNA	12.681
up	JX961639.1	heat shock protein 70 (Hsp70) mRNA, complete cds	44.783

Table S5. Genes differentially expressed in Medfly L3 in Response to heat treatment. T1 vs T0 are genes differentially expressed immediately after heat treatment. T2 vs T0 are genes differentially expressed 2 hrs after the heat treatment.

	Gene names	Gene Description	Fold change
**T1 VS T0**			
down	NCBI2_gene11747	transmembrane 147	0.001
down	NCBI2_gene571	tigger transposable element-derived 1-like	0.003
down	CURATIONS_gene1520	NPC2 homolog	0.038
down	NCBI2_gene8790	elongation of very long chain fatty acids AAEL008004	0.093
down	NCBI2_gene9866	heat shock 68	0.216
down	NCBI2_gene11774	heat shock 70	0.276
down	NCBI2_gene4888	heat shock 27	0.348
up	NCBI2_gene5031	MAK16 homolog	6.256
up	NCBI2_gene5376	DNA-binding RFX2	28.37
up	NCBI2_gene486	nucleolar 16	375.49
up	NCBI2_gene6786	OCIA domain-containing 1	402.683
up	NCBI2_gene5687	leucine-rich repeat 1	412.078
up	NCBI2_gene1022	PREDICTED: uncharacterized protein LOC101459691	461.376
up	NCBI2_gene3359	FAM92A1 isoform X1	548.808
up	NCBI2_gene5077	thiamin pyrophosphokinase 1 isoform X1	600.381
T2 vs T0			
down	CURATIONS_gene143	trypsin theta-like	0.001
down	CURATIONS_gene976	PREDICTED: uncharacterized protein LOC101449232	0.002
down	NCBI2_gene6399	actin cytoskeleton-regulatory complex PAN1	0.003
down	NCBI2_gene3777	diphthamide biosynthesis 1	0.062
down	NCBI2_gene7316	PREDICTED: uncharacterized protein Tm205 isoform X1	0.1

down	NCBI2_gene2024	succinate dehydrogenase assembly factor mitochondrial-like	0.105
down	NCBI2_gene11925	sarcoplasmic calcium-binding	0.123
down	NCBI2_gene9329	SERAC1 isoform X1	0.125
down	NCBI2_gene4895	heat shock 23-like	0.129
down	NCBI2_gene3966	tribbles homolog 3	0.143
down	NCBI2_gene3733	enhancer of split malpha	0.195
down	NCBI2_gene9866	heat shock 68	0.196
down	NCBI2_gene11551	msta-like isoform X1	0.208
down	NCBI2_gene8818	PREDICTED: uncharacterized protein LOC101450552	0.221
down	NCBI2_gene3838	PREDICTED: uncharacterized protein LOC101463046	0.231
down	NCBI2_gene4888	heat shock 27	0.261
down	NCBI2_gene1851	rho GTPase-activating gacZ	0.267
down	NCBI2_gene5754	trypsin I-P1	0.299
down	NCBI2_gene8429	la-related 1	0.309
down	CURATIONS_gene1138	eukaryotic translation initiation factor 4E-binding 3	0.318
down	NCBI2_gene12031	PREDICTED: uncharacterized protein LOC105665410	0.322
down	NCBI2_gene3577	PREDICTED: uncharacterized protein LOC101450402	0.342
down	CURATIONS_gene25	E3 ubiquitin- ligase MYLIP	0.344
down	NCBI2_gene3786	PREDICTED: uncharacterized protein LOC101455842	0.348
down	NCBI2_gene10941	mediator of RNA polymerase II transcription subunit 15a	0.349
down	NCBI2_gene1132	PREDICTED: protein scylla	0.357
down	NCBI2_gene4892	homeobox 5-like	0.366
down	NCBI2_gene10245	PREDICTED: uncharacterized protein LOC101462416	0.373
down	NCBI2_gene8474	CRISP Allergen PR- partial	0.376

down	NCBI2_gene5929	homeobox 13	0.386
down	NCBI2_gene949	teneurin-a isoform X2	0.397
down	NCBI2_gene4874	dnaJ homolog 1	0.399
down	NCBI2_gene6019	hairy isoform X1	0.400
down	NCBI2_gene11774	heat shock 70	0.413
down	NCBI2_gene9072	PREDICTED: uncharacterized protein LOC105665134	0.414
down	NCBI2_gene7498	PREDICTED: uncharacterized protein LOC101448507	0.436
down	NCBI2_gene5407	L-lactate dehydrogenase isoform X1	0.458
down	NCBI2_gene1482	serine-rich adhesin for platelets	0.492
up	NCBI2_gene1887	heat shock 83	2.353
up	NCBI2_gene614	PREDICTED: uncharacterized protein LOC101454195	2.665
up	NCBI2_gene3965	ras guanine nucleotide exchange factor P	2.774
up	NCBI2_gene5268	alpha beta hydrolase domain-containing 17B	2.860
up	NCBI2_gene9930	max X isoform X1	2.898
up	NCBI2_gene3544	myrosinase 1-like	3.088
up	CURATIONS_gene913	phenoloxidase 2	4.234
up	NCBI2_gene6865	water dikinase	6.101
up	NCBI2_gene4671	G patch domain-containing 4	11.87
up	NCBI2_gene11689	PREDICTED: uncharacterized protein LOC101449312	11.893
up	NCBI2_gene5376	DNA-binding RFX2	19.467
up	NCBI2_gene9240	PREDICTED: uncharacterized protein LOC101450980	41.775
up	NCBI2_gene1683	SET and MYND domain-containing 5	284.78
up	NCBI2_gene1893	PREDICTED: uncharacterized protein LOC101449609	300.041
up	NCBI2_gene8107	PREDICTED: uncharacterized protein LOC101461515	336.665
up	NCBI2_gene5687	leucine-rich repeat 1	339.021

up	NCBI2_gene4225	nuclease HARBI1 isoform X4	350.206
up	NCBI2_gene3359	FAM92A1 isoform X1	361.211
up	NCBI2_gene1865	cytochrome c oxidase assembly COX16 mitochondrial	365.767
up	NCBI2_gene1022	PREDICTED: uncharacterized protein LOC101459691	368.437
up	JAMg2_evm.TU.NW_004523968.1.49	CCR4-NOT transcription complex subunit 10 isoform X1	388.743
up	NCBI2_gene161	centrosomal of 131 kDa isoform X1	393.487
up	NCBI2_gene2900	ADP-ribosylation factor 2	431.951
up	NCBI2_gene486	nucleolar 16	437.001
up	CURATIONS_gene1637	odorant binding	509.916
up	NCBI2_gene6786	OCIA domain-containing 1	545.353
up	NCBI2_gene10413	PREDICTED: uncharacterized protein LOC101451833	549.258
up	NCBI2_gene5221	PREDICTED: uncharacterized protein LOC101460304	554.97
up	NCBI2_gene1590	FERM domain-containing 5	741.165
up	NCBI2_gene5077	thiamin pyrophosphokinase 1 isoform X1	767.884
